# A New Hyperchaotic 4D-FDHNN System with Four Positive Lyapunov Exponents and Its Application in Image Encryption

**DOI:** 10.3390/e24070900

**Published:** 2022-06-29

**Authors:** Zefei Liu, Jinqing Li, Xiaoqiang Di

**Affiliations:** 1 School of Computer Science and Technology, Changchun University of Science and Technology, Changchun 130022, China; 2019100629@mails.cust.edu.cn (Z.L.); dixiaoqiang@cust.edu.cn (X.D.); 2 Jilin Province Key Laboratory of Network and Information Security, Changchun 130033, China; 3 Information Center, Changchun University of Science and Technology, Changchun 130022, China

**Keywords:** 4D-FDHNN, dynamic analysis, fractal-like scrambling, Hilbert curve, dynamic random diffusion, image encryption

## Abstract

In this paper, a hyperchaotic four-dimensional fractional discrete Hopfield neural network system (4D-FDHNN) with four positive Lyapunov exponents is proposed. Firstly, the chaotic dynamics’ characteristics of the system are verified by analyzing and comparing the iterative trajectory diagram, phase diagram, attractor diagram, 0-1 test, sample entropy, and Lyapunov exponent. Furthermore, a novel image encryption scheme is designed to use the chaotic system as a pseudo-random number generator. In the scenario, the confusion phase using the fractal idea proposes a fractal-like model scrambling method, effectively enhancing the complexity and security of the confusion. For the advanced diffusion phase, we proposed a kind of Hilbert dynamic random diffusion method, synchronously changing the size and location of the pixel values, which improves the efficiency of the encryption algorithm. Finally, simulation results and security analysis experiments show that the proposed encryption algorithm has good efficiency and high security, and can resist common types of attacks.

## 1. Introduction

With the development of social networks and multimedia platforms, massive amounts of information are exposed under the open network, so data protection is extremely urgent. Because an image is one of the main media types of data transmission, the security of image data protection has become a hot topic for scholars. Image encryption is an effective way to protect image information. In recent years, a large number of excellent image encryption algorithms have emerged, constantly promoting the development and progress of image encryption.

The chaotic system has been widely used in the field of image encryption as a pseudo-random number generator due to its unique advantages of extreme sensitivity to initial parameters and unpredictability. Maria Munoz-Guillermo [1] designed an encryption algorithm based on the q variant Logistic mapping, which greatly improves the security of the algorithm by expanding the key space and parameter range of the system. Wang et al. proposed a fractional one-dimensional chaotic map with a large chaotic space and designed a real-time image encryption scheme based on the chaotic map [2]. Subsequently, to further improve the chaotic characteristics, Wang et al. also proposed a random scrambled image encryption algorithm on the basis of a one-dimensional Logistic self-embedding chaotic system, which effectively improved the security and efficiency of the encryption algorithm [3]. In order to enhance the complexity of chaotic system, Zhu et al. artificially constructed a five-dimensional continuous hyperchaotic system [4]. The encryption algorithm adopted a DNA dynamic coding mechanism and classical scrambling diffusion structure. Although these designed schemes are effective, they are also accompanied by several problems. This is because some low-dimensional chaotic systems are proved to be easy to predict due to their simple structure, while high-dimensional chaotic systems have a large chaotic space, but also bring about the problem that the structure is too complex to be suitable for real-time image encryption. Inspired by this, this paper proposes a four-dimensional fractional-order discrete neural network chaotic system (4D-FDHNN), which has complex dynamic behavior within the parameter range and does not cause low efficiency, and at the same time meets the real-time requirements of image encryption.

Generally speaking, the image encryption algorithm includes scrambling and diffusion. In order to obtain better an encryption effect, most of the existing image encryption schemes are organically combined with DNA coding [5], S-box transformation [6], compressed sensing, bit level [7], semi-tensor product operation [8,9], fractal model [10], and other advanced technologies. Ayubi et al. proposed a digital image encryption algorithm based on a generalized chaotic game model [11]. Chaos game is a famous fractal, which acts as a pseudo-random number generator in the proposed encryption algorithm, with dynamic behavior and complete chaos characteristics. Xian et al. proposed a kind of fractal ordering matrix with fractal characteristics, and this new matrix cluster can effectively improve the security of encryption algorithm by scrambling images or information [12]. In order to avoid the security risks in data transmission and storage environment, Sun et al. combined fractal dictionary coding with Julia Set and designed a new compression encryption scheme [13], which make a breakthrough in time and key security. When encrypting color images, Duan et al. determined the structural complexity of the nonlinear fractal scrambling method directly from the plaintext image, making the algorithm sufficiently resistant to known-plaintext attacks and chosen-plaintext attacks [14]. Hasanzadeh et al. designed a color image encryption scheme based on a hyperchaotic system by combining fractal with S-box in order to further improve the security of the algorithm [15]. The scheme uses a Hilbert fractal structure S box for scrambling, and Chen hyperchaotic system for diffusion operation. Experimental results show that the algorithm has high security in key space and sensitivity. It can be seen that fractal is widely used in image encryption algorithm and has become one of the effective encryption technologies. Therefore, the algorithm proposed in this paper slightly changes the fractal model and applies it to the scrambling process, thus effectively improving the complexity and security of the scrambling stage.

It is worth noting that some existing encryption systems are still vulnerable to cracking due to insufficient consideration of the chaotic characteristics of the system and the lack of security of the algorithm itself in the scheme design [16]. Dhall et al. made a cryptographic analysis of the image encryption scheme proposed in literature [17] and found some problems and unenforceability in the encryption scheme. Finally, they improved the scheme effectively to improve the security of the algorithm. In order to avoid such a situation, this paper adopts the dynamic random diffusion method based on Hilbert curve in the diffusion process when designing the encryption algorithm. When changing the size of the pixel value, the pixel position also changes, which not only improves the efficiency of the algorithm, but also greatly improves the security of the algorithm.

The advent of the information explosion era means that information security has become a key issue. Since images contain a wide range of information, including personal information, medical privacy and confidential remote sensing data, it is particularly important to protect their security. In view of the above problems in image encryption technology and the inspiration of existing encryption technology, the main contributions of this paper are as follows:

1. A new hyperchaotic four-dimensional fractional-order discrete Hopfield neural network system (4D-FDHNN) is proposed. The Lyapunov analysis indicates that the system has four positive Lyapunov exponent values, so it is called a hyperchaotic system. It also has rich dynamic characteristics, which is in line with the needs of image encryption for pseudo-random number generators.
2. Through the study and analysis of the fractal concept, a fractal-like model scrambling method is designed. This method has excellent scrambling effect, and the whole pixel matrix can be confused only after scrambling, which makes the image lose readability. This increases the complexity and security of the scrambling stage.
3. A Hilbert dynamic random diffusion scheme is designed to change the position and size of pixels synchronously, which strengthens the diffusion performance and improves the efficiency of the algorithm.

The rest of this paper is organized as follows: in Section 2, the hyperchaotic system of 4D fractional order discrete neural network is introduced and its dynamic behavior is analyzed. In Section 3, a kind of fractal model scrambling method is designed. The entire encryption algorithm is shown in Section 4. Section 5 is the experimental results and safety analysis. Finally, the whole paper is summarized in Section 6.

## 2. Four-Dimensional Fractional-Order Discrete Hopfield Neural Network and Its Dynamic Analysis

### 2.1. 4D-FDHNN Chaotic System

Low-dimensional chaotic systems often face security risks due to their simple structure and insufficient chaotic characteristics. High-dimensional chaotic systems will have the problem of too long an iteration time. Therefore, we have made certain improvements on the basis of the three-neuron fractional-order discrete Hopfield neural network proposed by Chen Liping et al. [18,19,20]. It is extended to the four-dimensional model, thereby enhancing the chaotic characteristics of the system, which is more suitable for the needs of image encryption. The improved four-dimensional fractional-order discrete Hopfield neural network (4D-FDHNN) is as follows:

(1) $$\left\{\begin{array}{cc} x(n+1) & =x\left(n\right)+\frac{h^{v}}{\Gamma (1+v)}[-x\left(n\right)+2\sin\left(x\left(n\right)\right)+\sin\left(y\left(n\right)\right)\\ \; & -9\sin(z(n))+2\sin(w(n))]\\ y(n+1) & =y\left(n\right)+\frac{h^{v}}{\Gamma (1+v)}[-y\left(n\right)-9\sin\left(x\left(n\right)\right)+2\sin\left(y\left(n\right)\right)\\ \; & +\sin(z(n))-9\sin(w(n))]\\ z(n+1) & =z\left(n\right)+\frac{h^{v}}{\Gamma (1+v)}[-z\left(n\right)+\sin\left(x\left(n\right)\right)-9\sin\left(y\left(n\right)\right)\\ \; & +2\sin(z(n))+\sin(w(n))]\\ w(n+1) & =w\left(n\right)+\frac{h^{v}}{\Gamma (1+v)}[-w\left(n\right)-9\sin\left(x\left(n\right)\right)+\sin\left(y\left(n\right)\right)\\ \; & -9\sin(z(n))+2\sin(w(n))] \end{array}\right.$$

Among them, x,y,z,w are the state variables of the 4D-FDHNN chaotic system, *h* is the discrete step size, and *v* is the fractional order, $h\in R^{+}$,v∈(0,1), $\Gamma \left(x\right)=\int_{0}^{\infty }t^{x-1}e^{-t}\;dt$, which represents the gamma function. Next, the dynamic characteristics of the modified system will be analyzed.

### 2.2. Random Behavior Analysis

**Figure 1:**
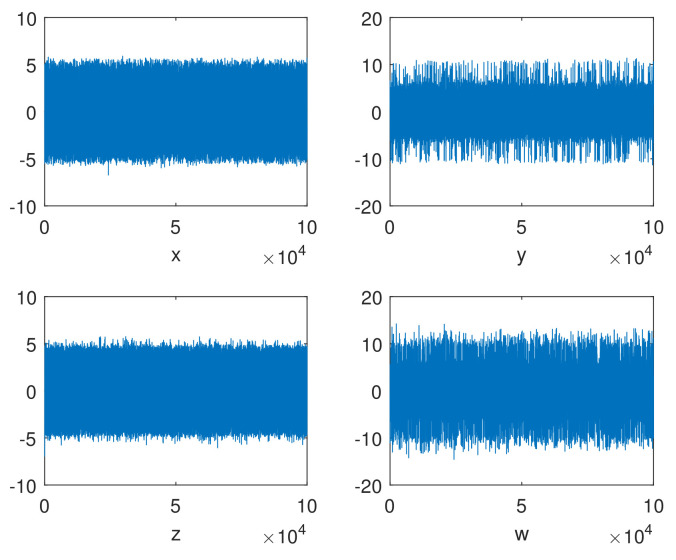
Random behaviors of the 4D-FDHNN chaotic system.

**Figure 2:**
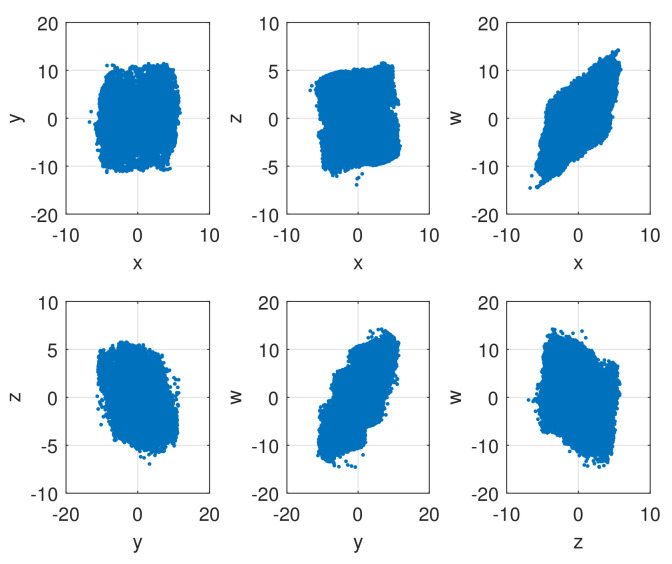
Phase diagrams of the 4D-FDHNN chaotic system for the initial value $\left(x_{0},y_{0},z_{0},w_{0}\right)=\left(0.08,0.8,-6.2,-0.62\right)$, fractional order v=0.6, and step size h=0.05 (x-y plane; x-z plane; x-w plane; y-z plane; y-w plane; z-w plane).

**Figure 3:**
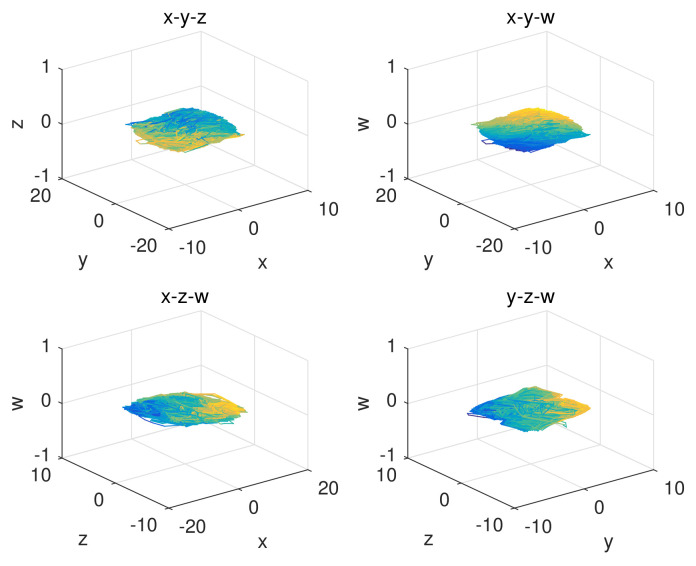
Attractor graph of the 4D-FDHNN chaotic system.

The random behaviors of the sequences x,y,z,w generated by a 4D-FDHNN chaotic system when $\left(x_{0},y_{0},z_{0},w_{0},h,v\right)$=(0.08,0.8,−6.2,−0.62,0.05,0.6) with $10^{5}$ iterations is illustrated in Figure 1. The horizontal axis represents the number of iterations, and the vertical axis is the value of the chaotic sequence. The graphs show that the values generated by the system are randomly distributed in the range of approximately −5 to 5 and −10 to 10. Furthermore, we can observe the phase diagram and the attractor diagram of the system. As shown in Figure 2, the phase diagram of 4D-FDHNN chaotic system is described in two-dimensional space. The attractor diagram of three-dimensional space is depicted in Figure 3. The phase diagram and the attractor diagram show that the trajectories eventually converge to a disorderly state rather than converging to an equilibrium point, whether the perturbations are small or large. It is thus clear that the 4D-FDHNN chaotic system exhibits good chaotic behavior.

### 2.3. 0-1 Test

**Figure 4:**
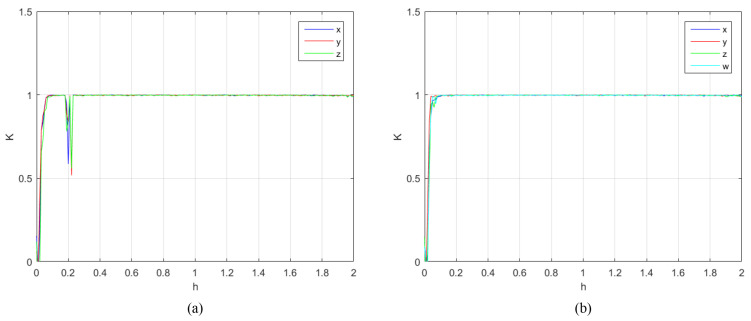
0-1 test results. (**a**) the 0-1 test of 3D-FDHNN with h∈(0,2),v=0.6; (**b**) the 0-1 test of 4D-FDHNN with h∈(0,2),v=0.6.

**Figure 5:**
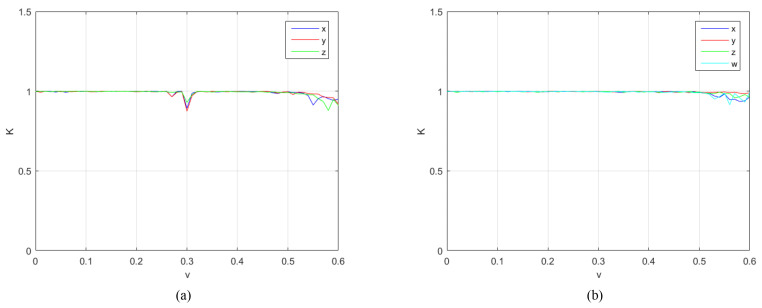
0-1 test results. (**a**) the 0-1 test of 3D-FDHNN with v∈(0,0.6),h=0.05; (**b**) the 0-1 test of 4D-FDHNN with v∈(0,0.6),h=0.05.

In order to further verify the chaotic characteristics of the proposed chaotic system, it is compared and analyzed with the 4D-FDHNN chaotic system. The 0-1 test is used to distinguish between regular and chaotic dynamics in deterministic dynamical systems [21]. It can measure the chaotic state of the time series, and the results obtained are close to 0 or 1, 0 corresponding to regular dynamics and 1 to chaotic dynamics [22]. We perform 0-1 tests using the calculations in the literature [22]. Figure 4 and Figure 5 depict the 0-1 test of two chaotic systems under the parameters h∈(0,2) and v∈(0,0.6), respectively. It can be clearly seen from the figure that the test results of the 4D-FDHNN chaotic system within the parameter range are basically close to 1, while the 4D-FDHNN chaotic system has a certain decline and fluctuation.

### 2.4. Sample Entropy Analysis

**Figure 6:**
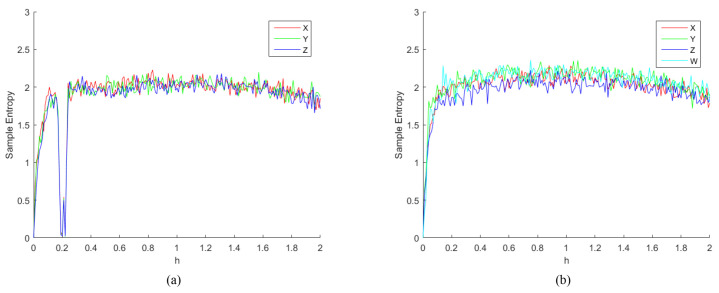
Sample Entropy. (**a**) the sample entropy of 3D-FDHNN with h∈(0,2); (**b**) the sample entropy of 4D-FDHNN with h∈(0,2).

**Figure 7:**
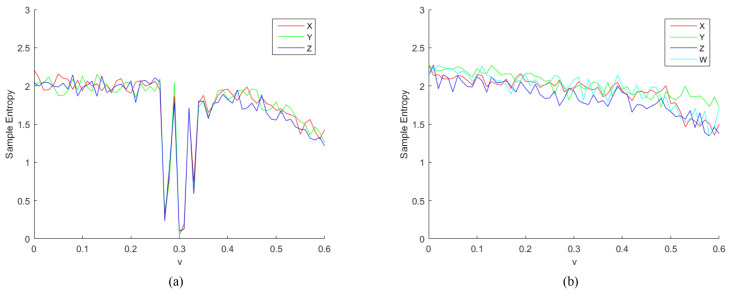
Sample Entropy. (**a**) the sample entropy of 3D-FDHNN with v∈(0,0.6); (**b**) the sample entropy of 4D-FDHNN with v∈(0,0.6).

Sample Entropy (SE) is derived from approximate entropy, which is a measure of the complexity of a time series [23]. SE is derived from approximate entropy and overcomes the problem that approximate entropy statistics can lead to inconsistent results, as proposed by Joshua et al. [24] in 2000. The complexity of the time series is measured by measuring the probability of generating a new pattern in the signal. The greater the probability of a new pattern, the greater the complexity of the sequence. In other words, the lower the value of sample entropy, the higher the self-similarity of the sequence. Conversely, the larger the value of sample entropy, the more complicated the sample sequence. We calculated the sample entropy using the method of literature [24] as shown in Figure 6 and Figure 7, which show the results of the sample entropy of the chaotic sequence generated by the two chaotic systems under the parameter h∈(0,2) and v∈(0,0.6). Obviously, the SE values of the 4D-FDHNN chaotic system fluctuate slightly around 2, while the three-neuron chaotic system performs unsatisfactorily at h∈(0,0.2) and v∈(0.25,0.35). Through the above analysis, the proposed 4D-FDHNN chaotic system has more complex dynamics and is suitable for the needs of image encryption.

### 2.5. Lyapunov Exponent Analysis

**Figure 8:**
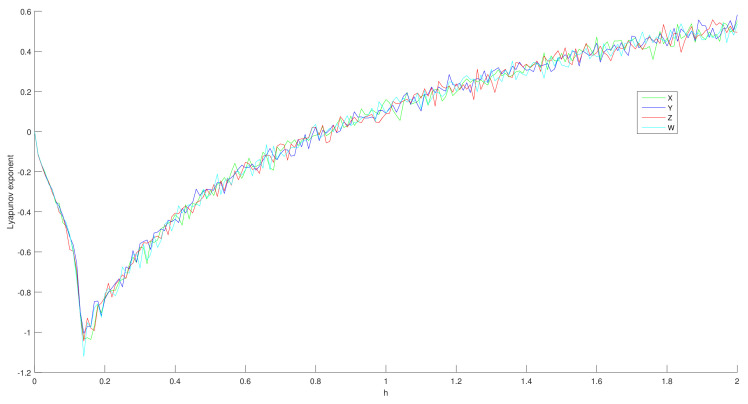
Lyapunov exponent.

Lyapunov exponent is a characteristic feature of chaotic systems that initially nearby trajectories separate exponentially in time. It is typically computed by following the linearization along a given reference trajectory. In ergodic systems, most trajectories will yield the same Lyapunov exponent, asymptotically for long times. The positive Lyapunov exponent is the source of the local instability of the chaotic attractor, which leads to the exponential separation of the two orbits generated by the two initial values with time, and since the system is dissipative, the chaotic system becomes locally unstable while the whole is stable [25]. Checking whether the Lyapunov exponent λ of the system is or is not positive can determine whether the system has chaotic motion. When λ<0, the system state tends to be stable and is not sensitive to the initial value; when λ>0, the system will eventually enter a chaotic state. Therefore, a chaotic system should have at least one positive λ. A system with two or more Lyapunov exponents is considered to be a hyperchaotic system. For discrete systems, the Lyapunov exponent is usually calculated by the eigenvalue method. We adopt a method based on QR orthogonal decomposition to calculate the Lyapunov exponents of the dynamic system proposed in this paper. The calculation method is shown in Equation (2) [26]:

(2) $$\left\{\begin{array}{c} \lambda_{1}=\lim_{T\to \infty }\frac{1}{T}\sum_{t=1}^{T}\ln\left|r_{1}^{\left(t\right)}\right|\\ \lambda_{2}=\lim_{T\to \infty }\frac{1}{T}\sum_{t=1}^{T}\ln\left|r_{2}^{\left(t\right)}\right|\\ \lambda_{3}=\lim_{T\to \infty }\frac{1}{T}\sum_{t=1}^{T}\ln\left|r_{3}^{\left(t\right)}\right|\\ \lambda_{4}=\lim_{T\to \infty }\frac{1}{T}\sum_{t=1}^{T}\ln\left|r_{4}^{\left(t\right)}\right| \end{array}\right.$$

where *T* is the times of orthogonal decompositions, and $r_{i}^{\left(t\right)}$ is the *i*-th (i=1,2,3,4) diagonal element corresponding to the upper triangular matrix obtained after the *t*-th orthogonal decomposition. This calculation method can avoid the error message of NaN or Inf due to too large number of iterations, and the problem of inaccurate calculation results caused by too few iterations. The calculation results are shown in Figure 8. It is not difficult to find that, when *h* is probably greater than 0.8, the four Lyapunov exponents all reach positive values. In order to more accurately represent the definite parameter range in which the Lyapunov exponent turns positive, the changes of the λ near 0 are listed in Table 1. According to the table and figure that, when h⩾0.88, $\lambda_{1},\lambda_{2},\lambda_{3},\lambda_{4}$ are all positive, i.e., the system has four positive Lyapunov exponents. Thus, 4D-FDHNN is a hyperchaotic system.

In conclusion, the proposed 4D-FDHNN shows good chaotic characteristics and is very suitable for the demand of pseudo random sequence generator for image encryption. Its application in image encryption will be studied, which follows.

## 3. Scrambling Method Based on a Fractal-like Model

Considering that the scrambling process of general algorithms is too simple and the overall security of the algorithm is insufficient [27], this section proposes a scrambling scheme based on a fractal-like model. This method improves the complexity and security of the scrambling phase. Before introducing specific methods, a brief explanation of fractals is given. The concept of fractal starts from the chaos game, and the basic theory of chaos game will be described below.

### 3.1. Chaos Game

**Figure 9:**
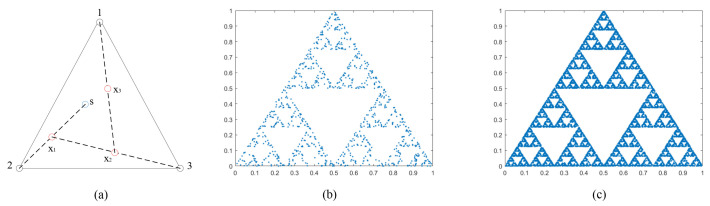
Chaos game with three bases. (**a**) the first three iterations, (**b**) 1000 iterations, (**c**) 10,000 iterations.

In mathematics, the term “chaos game” was originally proposed by Michael Barnsley [28], which represents a method of creating fractals by polygons and randomly selected initial points in them. Taking a triangle as an example, the rules for creating a point sequence of fractals through iteration are as follows: determine the three vertices of the triangle as bases and mark them as 1, 2, 3; select a point in the triangle as the initial point s; randomly select a base from 1, 2, and 3; the midpoint between the initial point s and the base is recorded as the new game point $x_{1}$; select a base randomly; the midpoint of $x_{1}$ and the selected base are marked as another new game point $x_{2}$, …, repeat the process until the number of iterations ends. An example of a simulated chaos game is shown in Figure 9. Figure 9a illustrates the first three iterations. The black circles indicate the position of three bases, the blue circles are the selected initial point, and the new game points generated by these three iterations are marked with red circles. Figure 9b,c show the effects of 1000 and 10,000 iterations, respectively.

The two-dimensional mathematical description of the Barnsley chaos game is as follows:

(3) $$\left\{\begin{array}{c} \mu_{n+1}=\mu_{n}+d\times \left(B\left[R\right]\left[1\right]-\mu_{n}\right)\\ \nu_{n+1}=\nu_{n}+d\times \left(B\left[R\right]\left[2\right]-\nu_{n}\right) \end{array}\right.$$

where μ,ν∈[0,1],R∈1,2,…,N (*N* is the number of bases), *d* is the distance ratio parameter in the game, the above example is the iteration result of d=0.5, and *B* is the two-dimensional array containing the bases coordinates of the chaos game.

**Figure 10:**
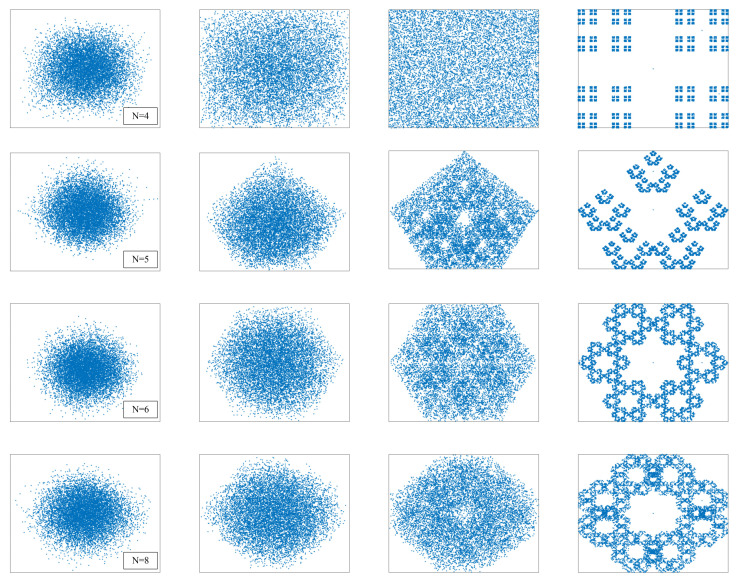
Fractal iteration results under different parameters. (Each column from left to right is d=0.15, d=0.35, d=0.5, and d=0.65, respectively.).

In order to observe the fractal phenomenon more clearly, fractal structures exhibited at different bases number N and different distance ratio d are simulated in Figure 10. The number of bases from top to bottom are N=4,5,6,8. The distance ratios d from left to right are 0.15, 0.35, 0.5, and 0.65, respectively. The corresponding bases’ coordinate matrices of the four different bases number are as follows:

   $B\left(N=4\right)=\left[\begin{array}{cc} 0 & 0\\ 0 & 1\\ 1 & 0\\ 1 & 1 \end{array}\right],B\left(N=5\right)=\left[\begin{array}{cc} 0 & 1/2\\ 1/2 & 1\\ 1 & 1/2\\ 1/4 & 0\\ 3/4 & 0 \end{array}\right]$

$B\left(N=6\right)=\left[\begin{array}{cc} 0 & 1/2\\ 1 & 1/2\\ 1/4 & 0\\ 3/4 & 0\\ 1/4 & 1\\ 3/4 & 1 \end{array}\right],B\left(N=8\right)=\left[\begin{array}{cc} 0 & 1/3\\ 0 & 2/3\\ 1/3 & 0\\ 2/3 & 0\\ 1/3 & 1\\ 2/3 & 1\\ 1 & 1/3\\ 1 & 2/3 \end{array}\right]$   

As we can find in the two figures above, the fractals have self-similarity. This is an interesting phenomenon. There is such a thing, no matter how you zoom in it, what you see is a cycle of similar patterns. That is, every detail of it looks almost exactly like the whole thing when blown up.

Fractal is produced by iteratively generating sequences in chaotic games. When specific parameter values are taken, sequences can also show chaotic characteristics [11]. After observing a series of iterations, at N=4, d=0.5, the new game points are almost evenly distributed in the entire space bounded by the rectangle. Similarly, the pixel space can also be viewed as a rectangular area, traversing each pixel value by iterating to create new game points, thus disrupting the entire pixel plane. Motivated by the above, this paper innovatively proposes a kind of fractal-like model scrambling method, which is introduced in the following section.

### 3.2. Fractal-like Model Scrambling Method

Scrambling is an important step in encryption algorithm. The complexity and unpredictability of the scrambling process are enhanced by using the fractal model, a scrambling method to make the encryption algorithm more secure. The procedure is given in detail:

Step 1. Choose a grayscale image IM with size of M×N as the original image.

**Figure 11:**
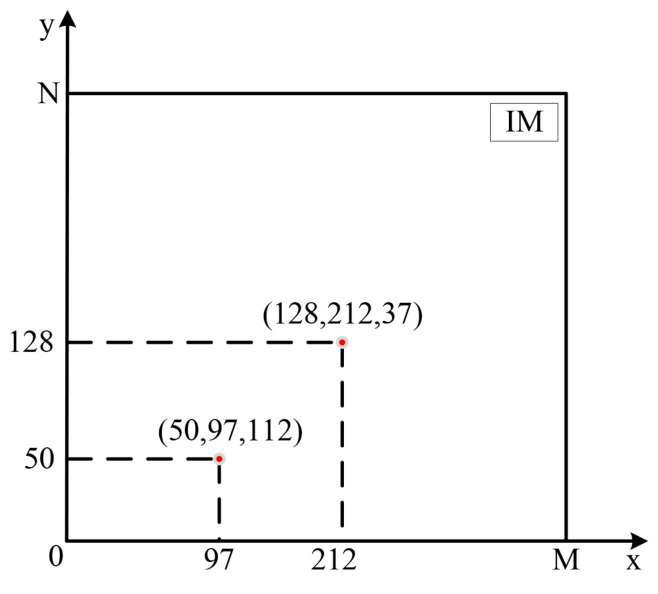
Pixel matrix mapped in a two-dimensional rectangular coordinate system.

Step 2. The pixel matrix represented by the original image IM is mapped in a two-dimensional rectangular coordinate system. As shown in Figure 11, (50,97,112) marked in red indicates that the pixel value of the image matrix at (50,97) is 112 in a two-dimensional rectangular coordinate system.

Step 3. The four corners of the two-dimensional pixel matrix IM are taken as the bases of the fractal-like model and recorded in the bases matrix *B*:

   $B\left(N=4\right)=\left[\begin{array}{cc} 1 & 1\\ 1 & N\\ M & 1\\ M & N \end{array}\right]$   

Step 4. For the sake of making each new game point determined can be located to a certain position in the pixel matrix, we make some modifications on the base of the original fractal model Equation (1). The structure of the fractal-like model is shown in Equations (4)–(6):

(4) $$\left\{\begin{array}{c} s_{1}=mod\left(floor\left(r_{1}\times 10^{12}\right),M\right)\\ s_{2}=mod\left(floor\left(r_{2}\times 10^{12}\right),N\right) \end{array}\right.$$

(5) $$\left\{\begin{array}{c} \mu_{1}=floor\left(s_{1}+d\times \left(B\left[R_{1}\right]\left[1\right]-s_{1}\right)\right)\\ \nu_{1}=floor\left(s_{2}+d\times \left(B\left[R_{1}\right]\left[2\right]-s_{2}\right)\right) \end{array}\right.$$

(6) $$\left\{\begin{array}{c} \mu_{n+1}=floor\left(\mu_{n}+d\times \left(B\left[R_{1}\right]\left[1\right]-\mu_{n}\right)\right)\\ \nu_{n+1}=floor\left(\nu_{n}+d\times \left(B\left[R_{1}\right]\left[2\right]-\nu_{n}\right)\right) \end{array}\right.$$

where n=1,2,…,M×N, $r_{1}=rand\left(\right)$, $r_{2}=rand\left(\right)$, which generates random number between 0 and 1. The random initial point s in the pixel matrix is generated from $r_{1}$ and $r_{2}$, and the coordinates are $\left(s_{1},s_{2}\right)$. d=0.5, $r_{1}$, $r_{2}$, *d* are used as the key. *R* is the bases random selection matrix, and one of the four bases is randomly selected for the new iterative process. The matrix R in this algorithm is calculated by a chaotic key stream generated by the chaotic system, and the specific calculation method is given a minute description in Section 4.2.

Step 5. As shown in Equation (6), initialize the value of all elements in matrix ISC to −1:

(7) ISC[i,j]=−1

where i=1,2,…,M, j=1,2,…,N.

Step 6: The matrix ISC is updated according to the pixel values in the image determined by the game point array generated in step 4, to reorder the original pixel matrix IM. The specific implementation process is shown in Algorithm 1. For the purpose of distinguishing the pixel positions that have been scrambled, the pixel value at the traversed position in IM is marked as −1.

Step 7: Insert the pixel values not traversed in the matrix IM into the position with the value of −1 in the matrix ISC sequentially. The pixel matrix SCR after the fractal-like model scrambling is obtained. For a clearer description, a pseudocode is given in Algorithm 2.

**Figure 12:**
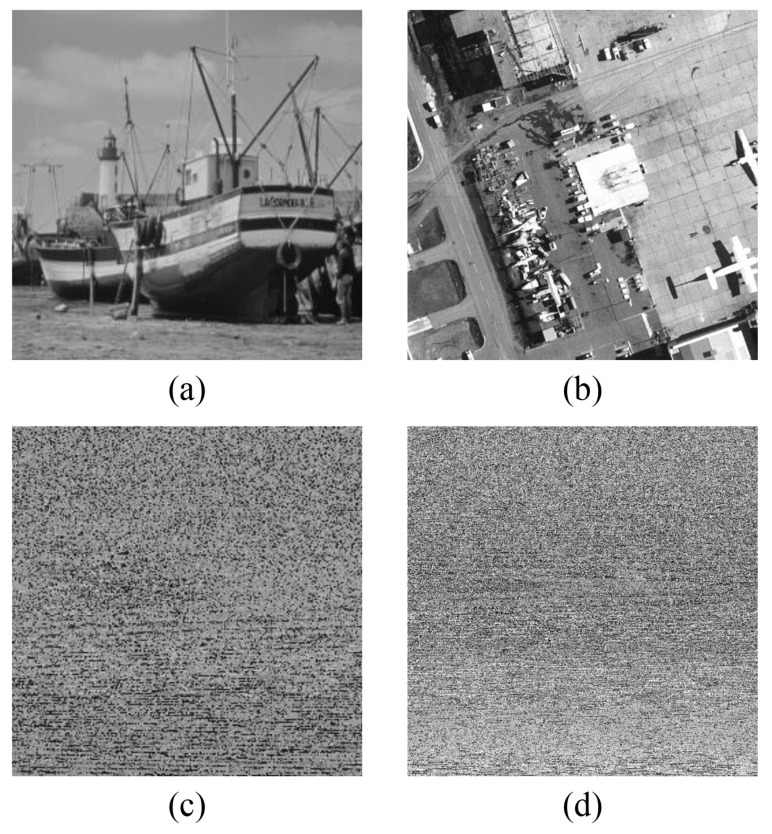
Scrambling effects of a fractal-like model. (**a**) original image “boat” (256×256); (**b**) original image “airfield” (512×512); (**c**) scrambled of (**a**); (**d**) scrambled of (**b**).

The above is the proposed fractal-like model scrambling method. Then, two pictures “boat” with size of 256×256 and “airfield” with size of 512×512 are used to test the scrambling effect. It can be clearly observed in Figure 12c,d that, only after the fractal-like model scrambling, the pixel values are fully disorganized, and no plaintext related information can be seen from the scrambled image. Consequently, the proposed scrambling method is effective.
**Algorithm 1** Fractal-like model scrambling I.**Input:** Original image IM, matrix ISC, Game point array $\left(\mu_{k},\nu_{k}\right)$
1:k = 1;2:**for** i=1 to *M* **do**3:   **for** j=1 to *N* **do**4:     **if** IM(i,j)=−1 **then**5:        IM(i,j) = $ISC(\mu_{k},\nu_{k})$;6:        $ISC(\mu_{k},\nu_{k})=-1$;7:        k=k+1;8:     **end if**9:   **end for**10:**end for**
**Output:** The updated matrix IM,ISC


**Algorithm 2** Fractal-like model scrambling II.**Input:** The updated matrix IM, ISC
1:$IM^{\prime }=$ reshape (IM,1,M×N);2:index=0;3:**for** i=1 to *M* **do**4:   **for** j=1 to *N* **do**5:     **if** ISC(i,j)=−1 **then**6:        **while** $\left(IM^{\prime }\left(index+1\right)=-1\right)$ **do**7:          index=index+1;8:        **end while**9:        $ISC\left(i,j\right)=IM^{\prime }\left(index+1\right)$;10:        index=index+1;11:     **end if**12:   **end for**13:**end for**14:SCR=ISC;
**Output:** Scrambling matrix SCR


## 4. Encryption Algorithm Based on a Fractal-like Model Scrambling and Hilbert Dynamic Random Diffusion

**Figure 13:**
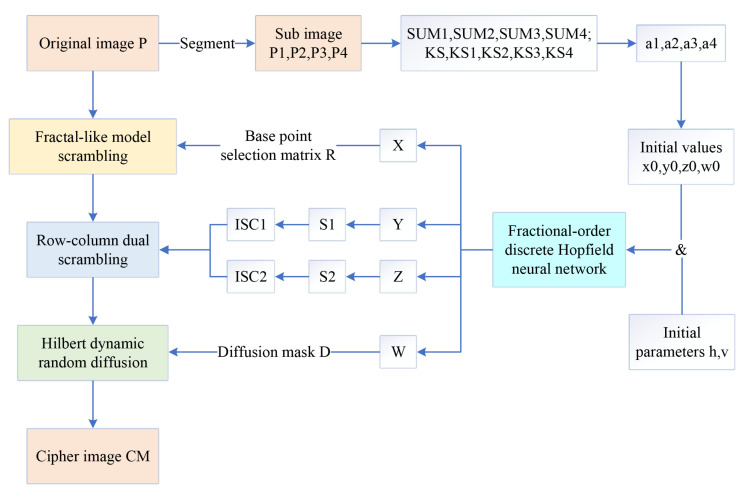
Encryption process.

The encryption algorithm includes four stages: chaotic key stream generation, fractal-like model scrambling, row-column dual scrambling, and Hilbert dynamic random diffusion. The encryption process is described in Figure 13, and the specific steps are as follows:

### 4.1. Generate Random Key Stream

Step 1. Divide the original image IM with size of M×N according to Equation (8) to obtain four sub-images $IM_{1}$, $IM_{2}$, $IM_{3}$, $IM_{4}$:

(8) $$\left\{\begin{array}{c} IM_{1}=IM\left(1:\frac{M}{2},;\right)\\ IM_{2}=IM\left(\frac{M}{2}+1,:\right)\\ IM_{3}=IM\left(:,1:\frac{N}{2}\right)\\ IM_{4}=IM\left(:,\frac{N}{2}+1:N\right) \end{array}\right.$$

Step 2. The sum of pixel values $SUM_{1},SUM_{2},SUM_{3}$ and $SUM_{4}$ of image $IM_{1},IM_{2},IM_{3},IM_{4}$, as well as their information entropy $KS,KS_{1},KS_{2},KS_{3},KS_{4}$ are calculated respectively, as shown in Equations (9) and (10):

(9) $$\left\{\begin{array}{c} SUM_{1}=sum\left(IM_{1}\left(:\right)\right)\\ SUM_{2}=sum\left(IM_{2}\left(:\right)\right)\\ SUM_{3}=sum\left(IM_{3}\left(:\right)\right)\\ SUM_{4}=sum\left(IM_{4}\left(:\right)\right) \end{array}\right.$$

(10) $$\left\{\begin{array}{c} KS=\sum_{i=0}^{2^{L}-1}p\left(x_{i}\right)\log_{2}\frac{1}{p(x_{i})}\\ KS_{1}=\sum_{i=0}^{2^{L}-1}p\left(x1_{i}\right)\log_{2}\frac{1}{p(x1_{i})}\\ KS_{2}=\sum_{i=0}^{2^{L}-1}p\left(x2_{i}\right)\log_{2}\frac{1}{p(x2_{i})}\\ KS_{3}=\sum_{i=0}^{2^{L}-1}p\left(x3_{i}\right)\log_{2}\frac{1}{p(x3_{i})}\\ KS_{4}=\sum_{i=0}^{2^{L}-1}p\left(x4_{i}\right)\log_{2}\frac{1}{p(x4_{i})} \end{array}\right.$$

where *L* is the gray level of the pixel, $p(x_{i})$, $p(x1_{i})$, $p(x2_{i})$, $p(x3_{i})$, and $p(x4_{i})$ represent the probability of the pixel value *i* in the image, respectively.

Step 3. The key stream generation process embeds parameters related to plaintext, which can effectively resist common known/chosen plaintext attacks. We use plaintext related pixel information $SUM_{1}$, $SUM_{2}$, $SUM_{3}$, $SUM_{4}$, and information entropy $KS,KS_{1},KS_{2},KS_{3},KS_{4}$ to generate the initial value of the chaotic system. The method is given in Equation (11). In this way, when inputting different original images for encryption, the system will generate completely different random key streams. This greatly enhances the security of the algorithm:

(11) $$\left\{\begin{array}{c} x_{0}=a+\frac{mod(floor\left(SUM_{1}+KS_{1}\times 10^{7}\right),M)}{10\times (M+N)}\\ y_{0}=b+\frac{mod(floor\left(SUM_{2}+KS_{2}\times 10^{7}\right),M)}{10\times (M+N)}\\ z_{0}=c+\frac{mod(floor\left(SUM_{3}+KS_{3}\times 10^{7}\right),M)}{10\times (M+N)}\\ w_{0}=d+\frac{mod(floor\left(SUM_{4}+KS_{4}\times 10^{7}\right),M)}{10\times (M+N)} \end{array}\right.$$

where mod() is remainder function, and floor() means round down function. $x_{0},y_{0},z_{0},w_{0}$ are the initial value of the 4D-FDHNN chaotic system, and a,b,c,d are the keys, which are set as (0.08, 0.8, −6.2, −0.62).

Step 4. Iterating the 4D-FDHNN chaotic system pp+M×N times with initial values ($x_{0},y_{0},z_{0},w_{0},h,v$). pp is an iterative parameter. In order to avoid the influence of instantaneous effect on the randomness of chaotic sequence, the former pp group values are discarded, and four groups of chaotic sequence X,Y,Z,W with the length of M×N are obtained. They are recorded as $X=x_{1},x_{2},x_{3},\ldots ,x_{MN}$, $Y=y_{1},y_{2},y_{3},\ldots ,y_{MN}$, $Z=z_{1},z_{2},z_{3},\ldots ,z_{MN}$, $W=w_{1},w_{2},w_{3},\ldots ,w_{MN}$. These pseudo random sequences will be used in the confusion and diffusion processes. The calculation method of pp is shown as follows:

(12) $$\left\{\begin{array}{c} p=ceil(KS\times \frac{1000}{\sqrt{M+N}})\\ pp=\left\{\begin{array}{cc} p & p⩾500\\ p+500 & p<500 \end{array}\right. \end{array}\right.$$

where ceil() stands for round up function.

### 4.2. Fractal-like Model Scrambling

The specific steps are explained in detail in Section 3.2, in which the chaotic sequence *X* is processed as Equation (13) to obtain the bases selection matrix *R*. After that, the scrambled matrix SCR is obtained:

(13) $$R=mod(ceil\left(mod\left(X,1\right)\times 10^{13}-KS\times 10^{9}\right),4)+1$$

### 4.3. Row-Column Dual Scrambling

To ensure the efficiency and enhance the scrambling effect, a row-column dual scrambling algorithm is added.

Step 1. As Equation (14), the chaotic sequence *Y*, *Z* is intercepted to obtain two sequences $S_{1}$ and $S_{2}$ with lengths of *M* and *N*, respectively:

(14) $$\left\{\begin{array}{c} S_{1}=Y\left(1:M\right)\\ S_{2}=Z\left(1:N\right) \end{array}\right.$$

Step 2. Arrange $S_{1}$ and $S_{2}$ in ascending order and record the index matrices $ID_{1}$ and $ID_{2}$ as follows:

(15) $$\left\{\begin{array}{c} ID_{1}=index\left(S_{1}\right)\\ ID_{2}=index\left(S_{2}\right) \end{array}\right.$$

Step 3. The index matrix $ID_{1}$ is used to perform further column scrambling on the pixel matrix SCR after fractal-like model scrambling. The pixel matrix $ISC_{1}$ after column scrambling is generated by Equation (16):

(16) $$ISC_{1}=SCR\left(:,ID_{1}\right)$$

Step 4. Perform row scrambling on the pixel matrix $ISC_{1}$ generated in the previous step with the index matrix $ID_{2}$. The pixel matrix $ISC_{1}$ after row scrambling is generated as Equation (17):

(17) $$ISC_{2}=ISC_{1}\left(ID_{2},:\right)$$

**Figure 14:**
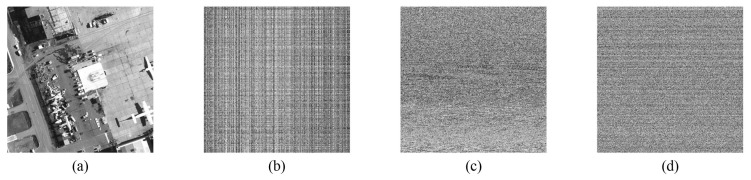
Comparison of scrambling effects. (**a**) original image “airfield”; (**b**) only row-column dual scrambling; (**c**) only fractal-like model scrambling; (**d**) combined scrambling (proposed).

The scrambling process is complete. Taking the 512×512 grayscale image “Airfield” as an example, a comparison diagram of the scrambling effect is depicted in Figure 14. Figure 14a is the original image “Airfield”, (b) shows the result of using only the row-column dual scrambling, (c) is the effect after using the fractal-like model scrambling, and (d) depicts the combination of these two methods. Visibly, the pixel information of the original image can be basically hidden after the fractal-like model scrambling. In addition, the scrambling effect has been further strengthened after the row-column dual scrambling. Almost no valid information is displayed in the ciphertext image.

### 4.4. Hilbert Dynamic Random Diffusion

#### 4.4.1. Hilbert Curve

There are many traversal scanning methods in two-dimensional space, the common ones are zigzag scanning, spiral scanning, raster scanning, Hilbert curve and so on [29]. These scanning methods can traverse every element in the matrix, and the traversal order is different. Therefore, it has been widely used in the scrambling process of image encryption. After the pixel matrix is traversed and reordered, the position of the pixels will be disrupted.

**Figure 15:**
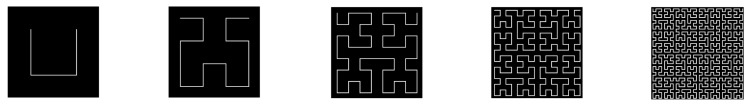
Hilbert curve of order 1–5.

The Hilbert curve is one of the scanning methods of the square array because it has a surjective effect in the array, that is, all the points in the matrix will be scanned when one traversal is completed. Figure 15 depicts the Hilbert curve of order 1, 2, 3, 4, and 5. Many scholars use this traversal method in the image scrambling process and have achieved good scrambling results. However, in this algorithm, we innovatively devote it to the diffusion process and randomly select the diffusion path according to the order of Hilbert traversal. The particular process of diffusion is described in the following section.

#### 4.4.2. Diffusion Process

Step 1. According to Equation (18), the chaotic sequence *W* is processed and its elements are mapped in the range of [0, 255] to obtain the sequence $W_{1}$ with the size of M×N:

(18) $$W_{1}=mod\left(floor\left(W\times 10^{13}\right),256\right)$$

Step 2. Transform $W_{1}$ into diffusion mask D with size of M×N, as follows:

(19) $$D=reshape(W_{1},M,N)$$

Step 3. The specific description of the Hilbert dynamic random diffusion method is shown in Equation (20). $h_{1}$ and $h_{2}$ are position matrices, which represent the horizontal and vertical coordinates of the Hilbert curve, respectively. After diffusion, the position of the pixel and the size of the pixel value change synchronously, and the security of the algorithm is enhanced. The final ciphertext image is CM.

(20) $$\left\{\begin{array}{cc} CM(h_{1}\left(1,1\right),h_{2}\left(1,1\right)) & =ISC_{2}\left(1,1\right)⨁D\left(1,1\right)\\ CM(h_{1}\left(1,j\right),h_{2}\left(1,j\right)) & =mod(ISC_{2}\left(1,j\right)+CM\left(h_{1}\left(1,j-1\right),h_{2}\left(1,j-1\right)\right),256)⨁D\left(1,j\right)\\ CM(h_{1}\left(i,1\right),h_{2}\left(i,1\right)) & =mod(ISC_{2}\left(i,1\right)+CM\left(h_{1}\left(i-1,1\right),h_{2}\left(i-1,1\right)\right),256)⨁D\left(i,1\right)\\ CM(h_{1}\left(i,j\right),h_{2}\left(i,j\right)) & =mod(ISC_{2}\left(i,j\right)+CM\left(h_{1}\left(i,j-1\right),h_{2}\left(i,j-1\right)\right)\\ & +CM(h_{1}\left(i-1,j\right),h_{2}\left(i-1,j\right)),256)⨁D\left(i,j\right) \end{array}\right.$$

where i=2,3,…,M, j=2,3,…,N.

## 5. Experimental Results and Safety Analysis

### 5.1. Simulation Results

**Figure 16:**
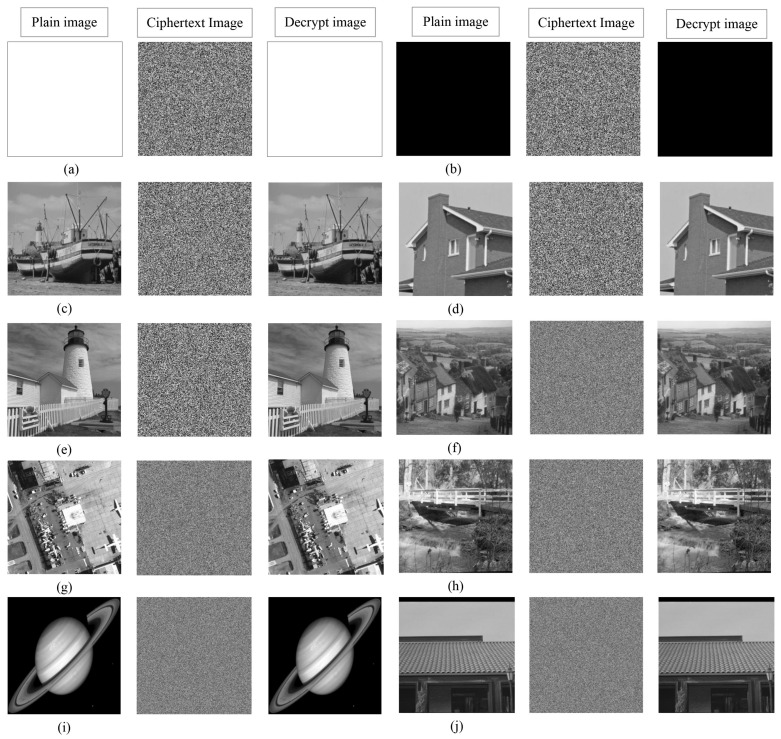
Encryption and decryption effects. (**a**–**e**) show the images with size of 256×256: “boat256”, “house256”, “kod256”, “all-white”, “all-black”. (**f**–**h**) are the images with size of 512×512: “hill512”, “airfield512”, “bridge512”. (**i**,**j**) are the images with the size of 1024×1024: “saturn1024”, “tile roof1024”.

For testing the encryption effect of the proposed algorithm, a simulation experiment was completed on the MATLAB 2015b platform. The computer environment is equipped with Intel(R) Core(TM)i7-6500U CPU@2.50 GHz, 8.00 GB RAM, and Windows 10 operating system. Select 10 grayscale images with sizes of 256×256, 512×512, and 1024×1024 from the standard database (https://ccia.ugr.es/cvg/dbimagenes/ accessed on 13 March 2014) to carry out the simulation experiment of encryption and decryption. The experimental results are shown in Figure 16. From the ciphertext image, it can be found that the encrypted images are noise-like and without any visible information. After decryption, the plain images can be completely restored without pixel loss. It is worth noting that the proposed algorithm is equally effective for all black and white images.

### 5.2. Key Security Analysis

#### 5.2.1. Key Space

A key space is a set of possible keys in an encryption system [30], indicating the range of key sizes. It is a key indicator used to detect the ability of an encryption algorithm to resist brute force cracking. The larger the key space, the stronger the ability of the algorithm to resist violent attacks, and the better the security performance of the algorithm. Generally, the key space greater than $2^{100}$ is considered to be able to resist the brute force attacks of modern computers [31]. The initial parameters ($x_{0},y_{0},z_{0},w_{0},h,v$) of the chaotic system, the plaintext related key KS, the random number $r_{1}$, $r_{2}$ of the base point random selection matrix, and the parameter d of the fractal-like model can be regarded as the security key. According to the IEEE standard, the accuracy of each initial key is $10^{15}$ [32]. With the premise of ensuring h⩾0.88, the key space of this algorithm is ${\left(10^{15}\right)}^{10}=10^{150}$. Because the system is chaotic when *h* is in this parameter range, beyond this range, the system will no longer have chaotic behavior. In addition, for special images of all-black or all-white, the initial parameter setting requires special attention to ensure that the system exhibits chaotic behavior within the parameter space, which is rarely used. The key space of this algorithm is much larger than $2^{100}$, hence, the key space of the algorithm is too large to resist brute force attacks.

#### 5.2.2. Key Sensitivity

**Figure 17:**
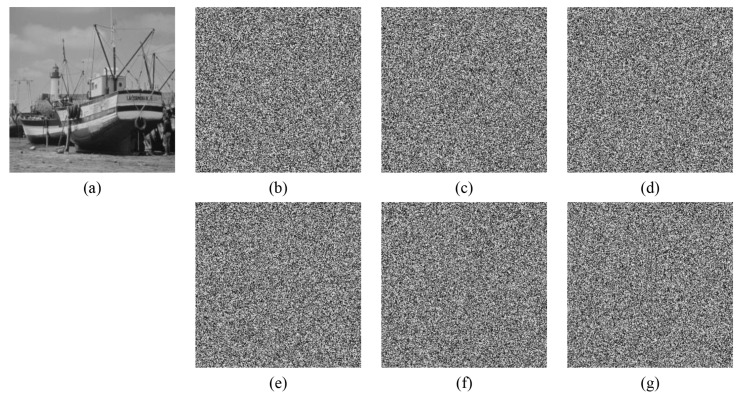
Key sensitivity of “boat256”. (**a**) The decrypted image with correct key; (**b**) the decrypted image with key $x_{0}+10^{-16}$; (**c**) the decrypted image with key $y_{0}+10^{-16}$; (**d**) the decrypted image with key $z_{0}+10^{-15}$; (**e**) the decrypted image with key $w_{0}+10^{-16}$; (**f**) the decrypted image with key $h+10^{-16}$; (**g**) the decrypted image with key $v+10^{-16}$.

**Figure 18:**
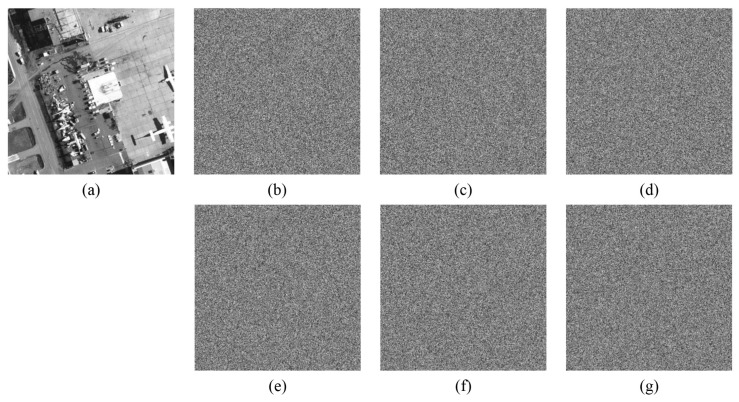
Key sensitivity of “airfield512”. (**a**) the decrypted image with a correct key; (**b**) the decrypted image with key $x_{0}+10^{-16}$; (**c**) the decrypted image with key $y_{0}+10^{-15}$; (**d**) the decrypted image with key $z_{0}+10^{-15}$; (**e**) the decrypted image with key $w_{0}+10^{-16}$; (**f**) the decrypted image with key $h+10^{-16}$; (**g**) the decrypted image with key $v+10^{-16}$.

**Figure 19:**
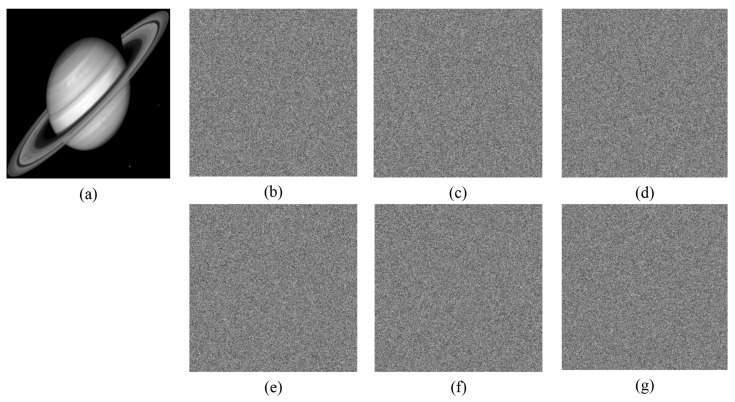
Key sensitivity of “saturn1024”. (**a**) the decrypted image with correct key; (**b**) the decrypted image with key $x_{0}+10^{-16}$; (**c**) the decrypted image with key $y_{0}+10^{-15}$; (**d**) the decrypted image with key $z_{0}+10^{-15}$; (**e**) the decrypted image with key $w_{0}+10^{-16}$; (**f**) the decrypted image with key $h+10^{-16}$; (**g**) the decrypted image with key $v+10^{-16}$.

Key sensitivity describes the influence of key change on decryption result. A good encryption algorithm should be sensitive enough to the key that it cannot recover the original image while the key changes only slightly. Figure 17, Figure 18 and Figure 19 show the decryption results of ’boat256’, ’airfield512’, and ’saturn1024’ of different sizes, respectively, when the correct key is used or only one key in ($x_{0},y_{0},z_{0},w_{0},h,v$) is changed. Obviously, a small change $10^{-15}$ or even $10^{-16}$ in a single key followed by decryption cannot restore the original image. This shows that the algorithm is more sensitive than ordinary algorithms that are only sensitive to $10^{-15}$ changes [5].

**Figure 20:**
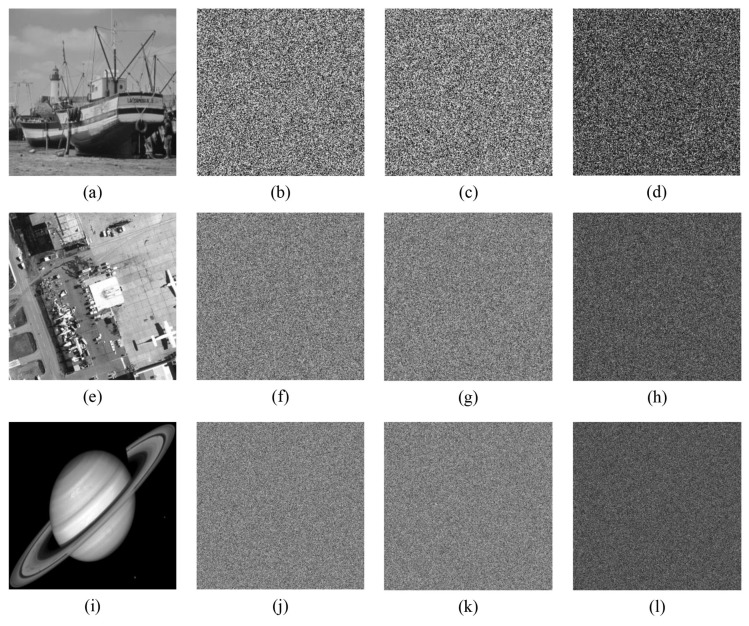
The difference between ciphertext images when the key changes slightly. (**a**) plain image “boat256”; (**b**) ciphertext Images with $x_{0}$; (**c**) ciphertext Images with $x_{0}+10^{-15}$; (**d**) the differences between (**b**,**c**); (**e**) plain image “airfield512”; (**f**) ciphertext Images with $y_{0}$; (**g**) ciphertext images with $y_{0}+10^{-15}$; (**h**) the differences between (**f**,**g**); (**i**) plain image “saturn1024”; (**j**) ciphertext Images with $z_{0}$; (**k**) ciphertext images with $z_{0}+10^{-15}$; (**l**) the differences between (**j**,**k**).

Furthermore, the impact of the small shift of the key on the generated ciphertext image is also tested. Taking ‘boat256’, ‘airfield512’, and ‘saturn1024’ as examples, the test results are displayed in Figure 20. By changing only $10^{-15}$ of the key $x_{0}$ of the image ‘boat256’, $y_{0}$ of the image ‘airfield512’, and $z_{0}$ of the image ‘saturn1024’, the differences between the two ciphertext images are 99.6139523%, 99.6292114%, and 99.5986938%, respectively. All of the above analysis proves that the key of this algorithm is highly sensitive.

### 5.3. NIST Randomness Tests

One of the essential criteria to examine the security of an image cipher is randomness. SP800-22 proposed by the “National Institute of Standards and Technology” (NIST) is one of the popularly used test suits. We have checked the randomness of the proposed scheme, and the results are shown in Table 2. The test suit contains various randomness tests based on different types of distribution such as normal distribution, chi-square distribution, half-normal distribution, etc. with different significant levels and *p*-values. It is usually considered that the test is successful when the *p*-value is greater than 0.01. The detailed *p*-values against different cipher images as listed in Table 2, and the scheme has passed all the tests confirming the randomness of the image cipher.

### 5.4. Statistical Analysis

#### 5.4.1. Histogram Analysis

**Figure 21:**
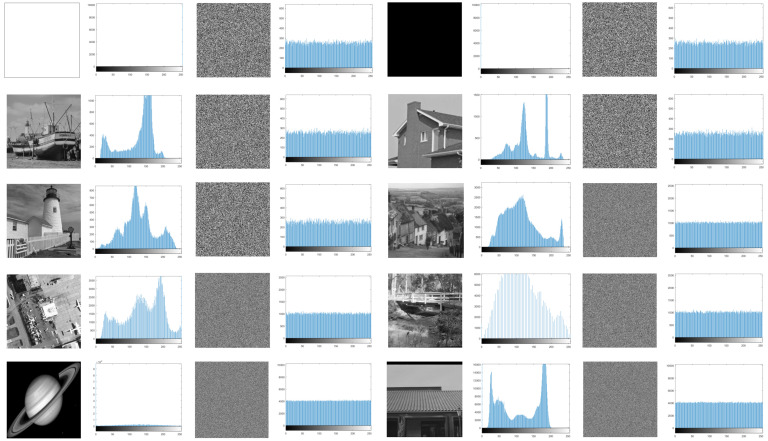
Histograms of plain images and their corresponding cipher images. Each plain image is followed by its histogram, the corresponding cipher image, and its histogram.

Histogram statistics is a significant graphic measure to evaluate the randomness and redundancy of intensity distribution, and it is used as a crucial observable index to evaluate the robustness of image encryption algorithm [33]. The flat histogram indicates that all gray levels occur the same numbers, the pixel distribution is uniform, and the randomness is high. Figure 21 tests the histograms of all image samples and their corresponding ciphertext images in this experiment. It can be clearly found that the histogram of the plain images fluctuates and is special uneven, while the ciphertext image is close to horizontal. This shows that the pixel values of the encrypted images are uniformly distributed, and the redundancies in the plain images are completely masked. An attacker cannot obtain any plaintext-related information from the histogram.

#### 5.4.2. Correlation Coefficient Analysis

Correlation coefficient is a statistical index that expresses the degree of correlation between variables. It is commonly used in image processing to study the relationship between two adjacent pixels. In a readable image, the correlation between adjacent pixels is usually high, with a correlation coefficient value close to 1. The smaller the correlation between two adjacent pixels, the closer the correlation coefficient is to 0, indicating the higher the security of the encrypted image. To investigate the obfuscation effect of encrypted images, the correlation between horizontal ($r_{h}$), vertical($r_{v}$) and diagonal($r_{d}$) adjacent pixels is tested respectively. The correlation coefficients of adjacent pixels are calculated as follows [34]:

(21) $$R_{pq}=\frac{cov(p,q)}{\sqrt{D(p)}\sqrt{D(q)}}$$

(22) $$\left\{\begin{array}{c} cov\left(p,q\right)=\frac{1}{n}\sum_{i=1}^{n}\left(p_{i}-E\left(p\right)\right)\left(q_{i}-E\left(q\right)\right)\\ E\left(p\right)=\frac{1}{n}\sum_{i=1}^{n}p_{i},D\left(p\right)=\frac{1}{n}\sum_{i=1}^{n}{\left(p_{i}-E\left(p\right)\right)}^{2}\\ E\left(q\right)=\frac{1}{n}\sum_{i=1}^{n}q_{i},D\left(q\right)=\frac{1}{n}\sum_{i=1}^{n}{\left(q_{i}-E\left(q\right)\right)}^{2} \end{array}\right.$$

where *n* is pair numbers of adjacent pixels, $p_{i}$ and $q_{i}$ are a pair of adjacent pixel values, E(p) is the mean of p, E(q) is the mean of *q*, D(p) is the variance of *p*, D(q) is the variance of *q*, and cov(p,q) represents the covariance of *p* and *q*. Randomly select 5000 pairs of adjacent pixels in each direction from the image samples used in this algorithm and their encrypted images. The correlation coefficients are calculated and compared with other advanced references, and the results are listed in Table 3. As can be seen from Table 3, the correlation between adjacent pixels in each direction in the plain images is strong, and the correlation coefficients are all close to 1. However, in the proposed encryption algorithm, the correlation coefficient of ciphertext image is closer to 0, which indicates that the correlation between adjacent pixels of ciphertext image can be ignored.

**Figure 22:**
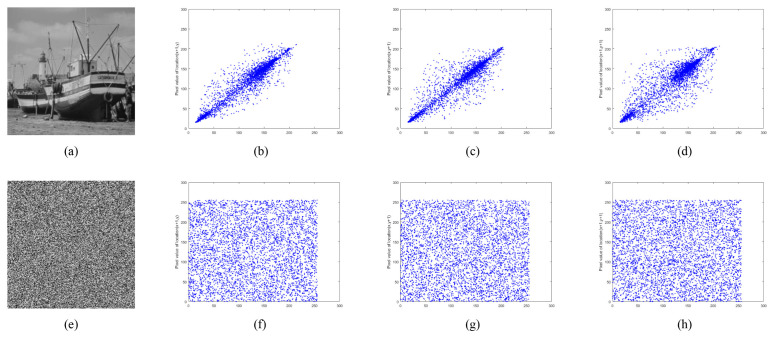
Adjacent pixel correlation of “boat256”. (**a**) plain image “boat256”; (**b**–**d**) correlation of adjacent pixels in the horizontal, vertical, and diagonal directions of (**a**); (**e**) ciphertext image of (**a**); (**f**–**h**) correlation of adjacent pixels in the horizontal, vertical, and diagonal directions of (**e**).

**Figure 23:**
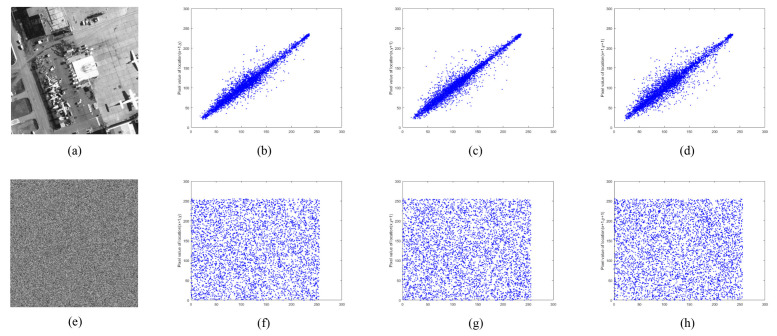
Adjacent pixel correlation of “airfield512”. (**a**) plain image “airfield512”; (**b**–**d**) correlation of adjacent pixels in the horizontal, vertical, and diagonal directions of (**a**); (**e**) ciphertext image of (**a**). (**f**–**h**) correlation of adjacent pixels in the horizontal, vertical, and diagonal directions of (**e**).

**Figure 24:**
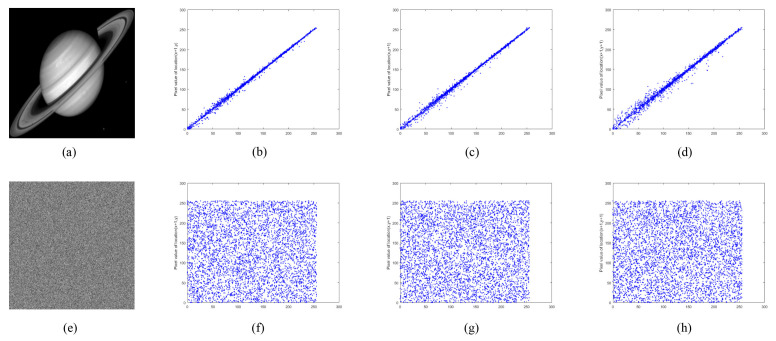
Adjacent pixel correlation of “saturn1024”. (**a**) plain image “saturn1024”; (**b**–**d**) correlation of adjacent pixels in the horizontal, vertical, and diagonal directions of (**a**); (**e**) ciphertext image of (**a**); (**f**–**h**) correlation of adjacent pixels in the horizontal, vertical, and diagonal directions of (**e**).

Figure 22, Figure 23 and Figure 24 show the distribution of adjacent pixels in “boat256”, “airfield512”, and “saturn1024”, where (b–d) are the distribution on horizontal, vertical, and diagonal of plain image, (f–h) are the distribution of ciphertext image in three directions. In (b–d), the pixels gather along the diagonal, and there is an obvious strong correlation between adjacent pixels in the plain image. However, in (f–h), the pixels are evenly distributed on the entire plane, indicating that the correlation of the ciphertext is greatly lessened. By reducing the correlation of adjacent pixels, the proposed encryption algorithm effectively avoids attackers from obtaining information from adjacent pixels when intercepting ciphertext images.

#### 5.4.3. Information Entropy Analysis

The concept of information entropy was first proposed by Shannon [36], which can be used to quantify information and reflect the random distribution of images. Its calculation formula is shown in Equation (23) [37]. The more disordered a system is, the higher its entropy and the more effort it takes for an attacker to crack it. In image processing, ciphertext images with higher entropy can resist entropy attack. For a 256-level gray image, the maximum value of information entropy is 8:

(23) $$H=\sum_{i=0}^{255}p\left(x_{i}\right)\log_{2}\frac{1}{p(x_{i})}$$

where $x_{i}$ represents the *i*-th pixel value, and $p(x_{i})$ represents the probability of pixel $x_{i}$. Table 4 is the calculation results and comparative analysis of the information entropy of the image sample in this experiment. The information entropy of ciphertext image is close to 8. Compared with other algorithms, the proposed algorithm has greater information entropy. This shows that the encrypted image is extremely random, which is difficult to crack.

### 5.5. Plain Image Sensitivity Analysis

Excellent encryption algorithms need to be able to resist differential attacks. In other words, it is sensitive enough to the plain image, and the small change of the original image will make the generated ciphertext image most different. Pixel change rate (NPCR) and uniform average change intensity (UACI) are two indicators commonly used to describe the ability of encryption algorithms to withstand differential attacks, and it is also possible to estimate the performance of the diffusion process [40]. NPCR is to calculate the percentage of differences numbers in the corresponding pixel values of two images in all pixels. UACI portrays the difference between all the corresponding pixels of two images. The ideal values of NPCR and UACI are 99.6094 and 33.4635, respectively. It can be calculated by Equations (24) and (25) [41]:

(24) $$\left\{\begin{array}{c} NPCR=\frac{\sum_{i,j}D\left(i,j\right)}{MN}\times 100D\left(i,j\right)=\left\{\begin{array}{cc} 0 & C\left(i,j\right)=C^{\prime }\left(i,j\right)\\ 1 & C\left(i,j\right)\neq C^{\prime }\left(i,j\right) \end{array}\right. \end{array}\right.$$

(25) $$UACI=\frac{\sum_{i,j}\left|C\left(i,j\right)-C^{\prime }\left(i,j\right)\right|}{MN}\times 100$$

where *M* and *N* represent the size of the image. *C* and $C^{\prime }$ are the normal ciphertext image and the ciphertext image obtained by changing one pixel of the plain image. NPCR and UACI values of “boat256”, “airfield512”, and “saturn1024” are tested and listed in Table 5. Compared with other algorithms, the results of the proposed algorithm are closer to the ideal value. Therefore, the algorithm can resist differential attacks and is sensitive to plain images.

### 5.6. Peak Signal-to-Noise Ratio

Peak signal-to-noise ratio (PSNR) is the most common and widely used objective evaluation index of image, which is used to measure the degree of image distortion. In the field of image encryption, the degradation degree of the encrypted image is reflected by calculating the PSNR of the plain image and the encrypted image. PSNR is defined by mean square error (MSE) and calculated by the following equation [43]:

(26) $$MSE=\frac{1}{MN}\sum_{i=0}^{M-1}\sum_{j=0}^{N-1}{\left[P\left(i,j\right)-C\left(i,j\right)\right]}^{2}$$

(27) $$PSNR=10\times \log_{1}0\left(\frac{{\left(MAX_{I}\right)}^{2}}{MSE}\right)$$

where *M* and *N* represent the size of the image. P(i,j), C(i,j) represent the pixel of the original image and the ciphertext image. $MAX_{I}$ is the maximum value of the pixel. Generally speaking, the larger MSE and the smaller PSNR indicate that more severe image distortion will affect the visual perception. Table 6 demonstrates the PSNR calculation results of “boat256”, “airfield512”, and “saturn1024”. The results display that the ciphertext image is seriously distorted, and no trace of the plain image can be seen.

### 5.7. Running Performance

#### 5.7.1. Computational Complexity

Computational complexity is also an important indicator for evaluating the quality of an algorithm. Even with high security, algorithms are not sufficient for practical applications. Therefore, the algorithm needs to ensure that the computational complexity is as low as possible on the basis of safety.

To compute the complications of performing the presented algorithm, the image size as M×N is taken into consideration. Let n indicate the quantity of pixels inside the image. The complexity of the presented algorithm can be determined by the following discussed operations. These operations consist of secret key generation, fractal-like model scrambling, row-column dual scrambling, and Hilbert dynamic random diffusion. The computational complexity of secret key generation is $O(n^{2})$, the complexity of fractal-like model scrambling is $O(3n^{2})$, the complexity of row-column dual scrambling is $O(n^{2})$, and the complexity of Hilbert dynamic random diffusion is $O(n^{2})$. Therefore, the total complexity of the proposed image encryption scheme is $O(5n^{2})$, which is much smaller than $O(14n^{2})$ of reference [33] and $O(78n^{2})$ of reference [44].

#### 5.7.2. Running Time

We tested the running time of all image samples in the experiment and performed comparative analysis. The results are listed in Table 7. After a large number of comparative analysis of encryption time with other relevant references, it can be noted that the proposed algorithm has a higher speed when encrypting images of the same size. Since the whole process is a symmetric algorithm, the time consumption of the decryption process is theoretically the same as that of encryption. This also verifies the superiority of the proposed algorithm.

### 5.8. Robustness Analysis

Cryptographic data are vulnerable to various threats when transmitted in open network space, such as noise pollution and pixel loss. Hence, the ideal encryption scheme should have strong robustness to resist different noise pollution and shear attacks [47].

#### 5.8.1. Noise Attack

**Figure 25:**
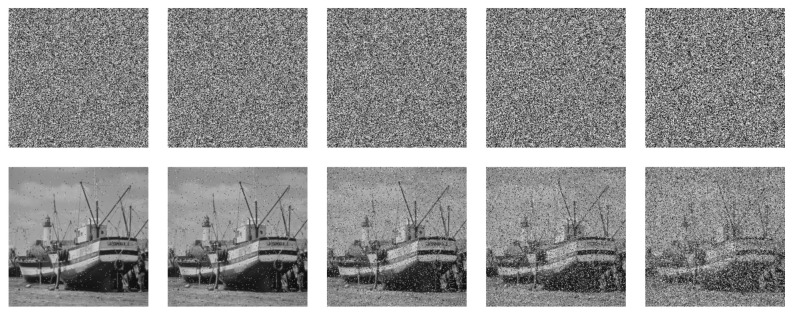
Salt and Pepper noise test of “boat256”. The first row from **left** to **right**: encrypted image with 0.5%, 1%, 5%, 10% and 20% Salt and Pepper noise added. The second row: decrypted image of the corresponding cipher image in the first row.

**Figure 26:**
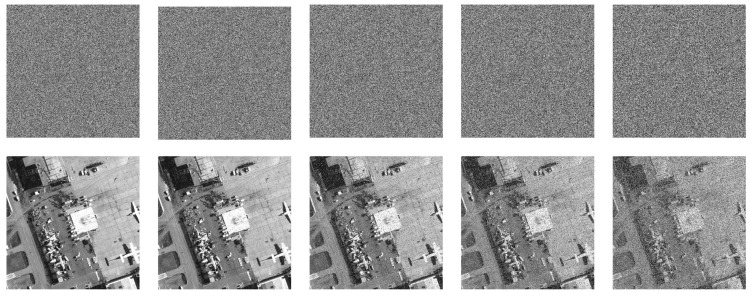
Salt and Pepper noise test of “airfield512”. The first row from **left** to **right**: encrypted image with 0.5%, 1%, 5%, 10% and 20% Salt and Pepper noise added. The second row: decrypted image of the corresponding cipher image in the first row.

**Figure 27:**
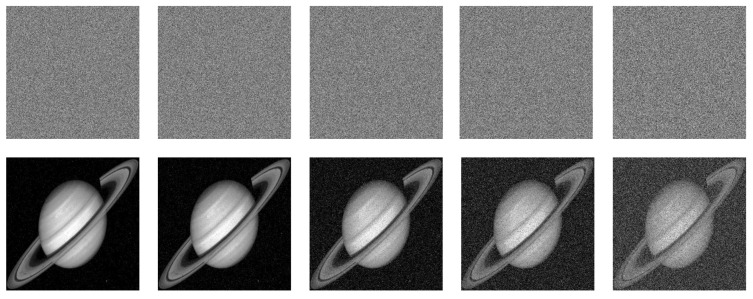
Salt and Pepper noise test of “saturn1024”. The first row from **left** to **right**: encrypted image with 0.5%, 1%, 5%, 10%, and 20% Salt and Pepper noise added. The second row: decrypted image of the corresponding cipher image in the first row.

**Figure 28:**
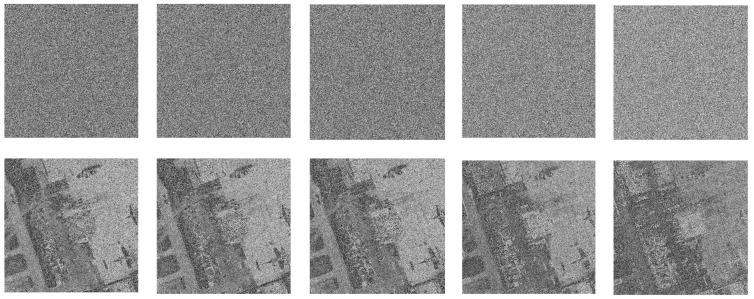
Gaussian noise test of “airfield512”. The first row from **left** to **right**: encrypted image with 0.1%, 0.5%, 1%, 5%, and 10% Gaussian noise added. The second row: decrypted image of the corresponding cipher image in the first row.

Figure 25, Figure 26 and Figure 27 depict the robustness analysis results of “boat256”, “airfield512”, and “saturn1024” against Salt and Pepper noise. The intensity of 0.5%, 1%, 5%, 10%, and 20% of Salt and Pepper noise were added to the cipher image. As we can see, the decrypted image is still visually valid. In addition, the ability of the algorithm to resist Gaussian noise is also investigated. Figure 28 shows the result of “airfield512” to Gaussian noise. We added 0.1%, 0.5%, 1%, 5%, and 10% Gaussian noise to the cipher image, which can basically resume the plain image after decryption. Experimental results certify that the proposed algorithm can recovery the image clearly despite different degrees of noise attacks.

#### 5.8.2. Clipping Attack

**Figure 29:**
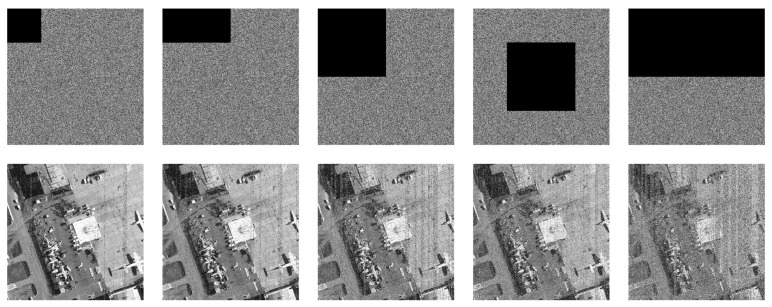
Clipping attack test of “airfield512”. The first row from **left** to **right**: encrypted image has been clipped 1/16, 1/8, 1/4, 1/4 (middle), and 1/2. The second row: decrypted image of the corresponding cipher image in the first row.

Apart from noise interference, the cipher image should also possess good anti-clipping attack performance [48]. Cut out the size of 1/16, 1/8, 1/4, and 1/2 of the ciphertext image “airfield512”, and the decryption results are portrayed in Figure 29. The main content of the image is still visible even if the clipping rate reaches 1/2. Consequently, the proposed algorithm can effectively resist clipping attack.

## 6. Conclusions

In this paper, based on four-dimensional fractional-order discrete Hopfield neural network (4D-FDHNN), an image encryption scheme is proposed. In order to strengthen the dynamic behavior of the chaotic system, the three-neuron chaotic system is improved and obtains a new 4D-FDHNN. The experimental results of dynamic analysis show that the system has good chaotic characteristics and is hyperchaotic. Then, 4D-FDHNN is applied to the design of image cryptography system. The feature information of the original image is extracted to generate the initial value of the chaotic system, and the plaintext correlation of the key is realized. The encryption step is performed with the chaotic key stream generated by the system. The scrambling process consists of two stages: fractal-like model scrambling and row-column double scrambling. In addition, then, the Hilbert dynamic random diffusion is performed to complete the entire encryption. Finally, a quantity of experimental performance analyses show that the proposed algorithm has the characteristics of large key space, high efficiency, and can resist various common attacks. In addition, the experimental results in this paper are only shown with a single-channel image as an example, and the proposed encryption scheme is also applicable to multi-channel images. The encryption process of multi-channel image can be completed by performing the proposed encryption method on the pixel matrix of each channel, and then synthesizing the generated ciphertext.

## Figures and Tables

**Table 1:** The key point of Lyapunov exponent turning into positive.

*h*	Lyapunov Exponent			
	$\lambda_{1}$	$\lambda_{2}$	$\lambda_{3}$	$\lambda_{4}$
0.84	−0.0211	−0.0078	−0.0561	−0.0022
0.85	−0.0094	0.0090	−0.0493	0.0177
0.86	0.00791	0.0330	0.0311	−0.0020
0.87	0.03733	−0.0059	−0.0027	0.0390
0.88	0.06598	0.0038	0.0751	0.0113

**Table 2:** NIST randomness tests for the proposed scheme and its comparison.

Image Size	256×256		512×512		1024×1024	
Tests	*p*-Value	Result	*p*-Value	Result	*p*-Value	Result
Approximate Entropy	0.921541	✓	0.948753	✓	0.85384	✓
Block Frequency	0.821153	✓	0.618297	✓	0.996283	✓
Cumulative Sums	0.42738	✓	0.023076	✓	0.125491	✓
FFT	0.705674	✓	0.552252	✓	0.196993	✓
Frequency	0.33783	✓	0.022113	✓	0.258306	✓
Linear Complexity	0.042136	✓	0.367285	✓	0.676756	✓
Longest Runs of 1 s	0.860484	✓	0.07015	✓	0.927182	✓
Non-overlapping Templates	0.500097	✓	0.353505	✓	0.351968	✓
Overlapping Templates	0.370476	✓	0.819045	✓	0.16745	✓
Random Excursions	0.841748	✓	0.050996	✓	0.140009	✓
Random Excursions Variant	0.101973	✓	0.589966	✓	0.340509	✓
Rank	0.615691	✓	0.727356	✓	0.036279	✓
Runs	0.858687	✓	0.561305	✓	0.853917	✓
Serial	0.283802	✓	0.403982	✓	0.70536	✓
Universal	0.806738	✓	0.698998	✓	0.259228	✓

**Table 3:** Correlation coefficients between adjacent pixels of the image.

Image	Direction	Plain Image	Cipher Image
boat256	$r_{h}$	0.9546	−0.0198
	$r_{v}$	0.9416	0.0046
	$r_{d}$	0.9074	−0.0026
house256	$r_{h}$	0.9686	0.0082
	$r_{v}$	0.9773	0.0001
	$r_{d}$	0.9514	−0.0221
kod256	$r_{h}$	0.9127	0.0002
	$r_{v}$	0.8748	0.0005
	$r_{d}$	0.8016	0.0179
hill512	$r_{h}$	0.9737	0.0087
	$r_{v}$	0.9710	−0.0008
	$r_{d}$	0.9520	0.0046
airfield512	$r_{h}$	0.9412	−0.0007
	$r_{v}$	0.9421	−0.0060
	$r_{d}$	0.9059	0.0077
bridge512	$r_{h}$	0.9229	−0.0078
	$r_{v}$	0.9413	0.0059
	$r_{d}$	0.8932	0.0014
saturn1024	$r_{h}$	0.9731	0.0024
	$r_{v}$	0.9900	−0.0001
	$r_{d}$	0.9669	0.0012
tile roof1024	$r_{h}$	0.9996	−0.0007
	$r_{v}$	0.9995	0.0000
	$r_{d}$	0.9987	−0.0035
Ref. [29]	$r_{h}$	0.8364	0.0069
	$r_{v}$	0.8848	0.0037
	$r_{d}$	0.8690	−0.0079
Ref. [35]	$r_{h}$	0.9356	0.0236
	$r_{v}$	0.9604	0.0235
	$r_{d}$	0.9116	0.0189

**Table 4:** Information entropy.

Algorithm	Image	Information Entropy
Proposed	boat256	7.9970
	house256	7.9970
	kod256	7.9964
	all-white	7.9975
	all-black	7.9975
	hill512	7.9994
	bridge512	7.9992
	airfield512	7.9992
	saturn1024	7.9998
	tile roof1024	7.9998
Ref. [38]	256×256	7.9899
	512×512	7.9914
	1024×1024	7.9919
Ref. [39]	256×256	7.9975

**Table 5:** Plain image sensitivity analysis.

Algorithm	NPCR	UACI
Proposed-boat256	99.6109	33.4645
Proposed-airfield512	99.6048	33.4054
Proposed-saturn1024	99.6036	33.4606
Ref. [35]-256×256	99.6277	33.5045
Ref. [35]-512×512	99.6025	33.4814
Ref. [35]-1024×1024	99.6233	33.4678
Ref. [38]-256×256	99.6002	33.5524
Ref. [38]-512×512	99.5937	33.4086
Ref. [38]-1024×1024	99.5991	33.4656
Ref. [42]	98.9874	33.2516

**Table 6:** PSNR and MSE results.

Index	MSE	PSNR
boat256	7697.5205	9.2673
airfield512	9310.7735	8.4409
saturn1024	15,142.9713	6.3287

**Table 7:** Running time analysis.

Algorithm	Image Size	Running Time (s)
Proposed-boat256	256×256	0.0806
Proposed-house256	256×256	0.0757
Proposed-kod256	256×256	0.0774
Proposed-hill512	512×512	0.3395
Proposed-airfield512	512×512	0.3494
Proposed-bridge512	512×512	0.3203
Proposed-saturn1024	1024×1024	1.3284
Proposed-tile roof1024	1024×1024	1.3175
	512×512	3.3833
Ref. [29]	256×256	0.4060
Ref. [32]	256×256	0.8158
Ref. [38]	256×256	0.459837
	512×512	1.769703
	1024×1024	2.700164
Ref. [45]	512×512	0.7738
Ref. [46]	256×256	0.256722
	512×512	0.620413
	1024×1024	2.895086

## Data Availability

The used test images are all included in the paper.
